# The Effects of Different Representations on Static Structure Analysis of Computer Malware Signatures

**DOI:** 10.1155/2013/671096

**Published:** 2013-08-01

**Authors:** Ajit Narayanan, Yi Chen, Shaoning Pang, Ban Tao

**Affiliations:** ^1^School of Computing and Mathematical Sciences, Auckland University of Technology, Auckland 1010, New Zealand; ^2^Department of Computing, Unitec Institute of Technology, Auckland 1025, New Zealand; ^3^National Institute of Information and Communications Technology, Tokyo 184-8795, Japan

## Abstract

The continuous growth of malware presents a problem for internet computing due to increasingly sophisticated techniques for disguising malicious code through mutation and the time required to identify signatures for use by antiviral software systems (AVS). Malware modelling has focused primarily on semantics due to the intended actions and behaviours of viral and worm code. The aim of this paper is to evaluate a static structure approach to malware modelling using the growing malware signature databases now available. We show that, if malware signatures are represented as artificial protein sequences, it is possible to apply standard sequence alignment techniques in bioinformatics to improve accuracy of distinguishing between worm and virus signatures. Moreover, aligned signature sequences can be mined through traditional data mining techniques to extract metasignatures that help to distinguish between viral and worm signatures. All bioinformatics and data mining analysis were performed on publicly available tools and Weka.

## 1. Introduction

If users do not have confidence that their machines will not be attacked when connected to the internet, major areas of computing will be constrained due to fear of denial of service and massive data fraud [1]. Symantec reported over 5 billion attacks in 2011, an 81% increase over 2010 [2]. Over 400 million new malware variants were identified that year alone. From a theoretical perspective, while virus detection is undecidable [3–5], it is still not known whether there exist algorithms that will take an arbitrary program or code and decide correctly whether it contains specific forms of malware [6]. This is not just because malware is behavioural (actions performed at run time) and hence characterized semantically [7], usually in the form of execution traces [8], control flow [9], and process calculi [10]. Rather, an essential aspect of viruses and worms is obfuscation through polymorphic and metamorphic mutation [11–13], that is, the ability to replicate with modification. While polymorphic mutation (payload algorithm is kept constant, but viral code is mutated) has led to computable detection in some cases [6, 14, 15], metamorphic mutation involves generating logically equivalent code with changes in program length and flow as well as data structures [16]. Because of increasing complexity of obfuscation as well as discovery of new types of malware (e.g., spyware, botnets), human experts are still required to implement the variety of polymorphic and metamorphic malware detection techniques currently known to exist [17–20]. This manual process leads to the use of “signatures” by antiviral software systems when scanning network packets or memory block hashes for contiguous appearance of key parts of malware code. This in turn leads to the situation where malware infections must occur first before solutions can be found and hence the threat to user confidence. 

Research has continued in static structure checking algorithms [21–24] despite current emphasis on semantic-based approaches. Static structure analysis can reveal deep structural similarities between superficially dissimilar sequences irrespective of control flow. Static checkers have faced problems in identifying complex obfuscation, however [25]. We have recently demonstrated a potential breakthrough in static approaches by using the ever-expanding base of already available hexadecimal signatures [26] for polymorphic and metamorphic malware. The key was to represent these signatures under an interpretation derived from biology: amino acids forming polypeptide sequences. After signature alignment using bioinformatics sequence alignment techniques involving substitution matrices derived from the large number of biosequence databases now available, static metasignatures for distinguishing between worms and viruses were extracted with high accuracy [27, 28]. However, there are some limitations to this work. 

Antiviral signatures can be calculated from a pattern of operations in the malware code or can represent the encryption algorithm used to hide the virus or worm. Signatures were originally and continue to be identified and calculated by human experts and are typically a sequence of hexadecimal numbers intended to uniquely identify viruses and worms. Automatic generation of signatures for new malware continues to be a difficult problem [29]. Such signatures can also be consistent for a “family” of viruses or worms that share parts of the code or have similar function and are essentially variants of each other. For instance, “Virus.Acad.Bursted.a” is a typical computer virus name that indicates the platform (Autocad, or “Acad”), the family (Bursted), and the variant “a”. Achieving consistency of signatures for members of the same family is especially important when dealing with polymorphic (the functional parts of the code are the same but hidden differently) and metamorphic (the function remains the same, but the code is altered with every replication) malware designed to avoid such signature detection [30, 31]. Due to the security dangers inherent in making the original malware code available for public dissemination, only signatures are made publicly available.

AVS scanners use a dictionary or library of signatures in a variety of different ways. For instance, for simple polymorphic malware detection, the hexadecimal representation of a signature can be used to match against incoming network packets containing bytes also represented in hexadecimal. This allows the AVS to check for contiguous similarities between parts of the signature and packet contents. For metamorphic and more complex polymorphic malware detection, increasingly sophisticated techniques must be used that allow for contiguous parts of the signature to be detected noncontiguously across different packets [32]. Signature detection through pattern matching is usually supported by other techniques, such as stateful monitoring, to minimize false positives and false negatives [33]. Malware writers adopt a variety of sophisticated techniques for avoiding detection. By the time a new variant is identified and signatures released, the infection may already have reached epidemic proportions [34]. One of the problems in applying automatic data mining techniques to static malware code directly, even if it is available, is the variable length of the code [35], since most data mining and other machine learning techniques assume fixed length sequences with a column representing measurements of the same variable across many samples. There is surprisingly little work reporting on the application of machine learning techniques to malware signature detection, mainly due to the problem of obtaining malware source code as well as the need to deal with variable length code to identify the critical parts of the code from which to derive signatures. Also, mining the signatures directly can lead to results that are difficult to interpret, since the hexadecimal signatures cannot always be mapped back to meaningful and individual operations in the source code (op code). The variable length of the malware code, the difficulty of legally obtaining source malware code for detailed analysis, and the lack of interpretability of results if hexadecimal signatures are used and the partially sequential aspects of the data all obstruct the use of machine learning techniques, thereby limiting their use in the urgent problem of finding automatic ways of generating static signatures.

Sequence analysis is used in biology to understand the relationship between two or more sequences (multiple sequence alignment) of genetic information, such as DNA or amino acids. There are databases of genetic information which are processed by string alignment algorithms to better understand the relationship between species and also determine the location of specific genes. In particular, sequence analysis and alignment can be used to identify conserved regions or motifs (regions of similarity) in biological data that identify common genes and shared ancestry as well as common structure and function of amino acid sequences [36]. One advantageous side effect of alignment methods is that variable length biological sequences can be converted into fixed length sequences through appropriate insertion and deletion techniques. Powerful data mining algorithms that assume fixed length sequences or patterns can then be applied to identify critical features that help to determine whether a sequence is malware or not. 

Sequence alignment techniques are not confined to biological sequences, however, and there have been applications of sequence alignment in linguistics [37] and marketing [38]. The first demonstration of multiple sequence alignment to malware signatures to identify motifs [39], or metasignatures, for families of computer viruses and worms demonstrated the feasibility of the approach. The signatures of 30 worms and 30 viruses were converted into amino acid residue representation using a random mapping (hex 1 became “A”, hex 2 “C”..). Since there are 20 amino acid residue characters, that left four spare amino acid residues. The amino acid W was used to represent gaps in the alignment of worms and viruses separately and the amino acid Y to represent gaps in the alignment when the aligned worms and viruses were jointly aligned to produce a common fixed length set of sequences. ClustalW [40] was used as the multiple alignment tool. The advantage of alignment was that initially fixed length signatures can be expanded to find common or conserved regions across families of viruses and worms separately. The length of expansion will vary between families so that the length of the aligned signatures of worms will almost certainly be different from aligned virus signatures. These separately aligned worm and virus signatures were then multiply aligned together into fixed length (but significantly longer) sequences that were annotated with a class value (“1” for worm, “0” for virus) for supervised learning. The doubly aligned sequences were in turn converted into decimal ASCII code (“A” became 66, “C” 67 … “Z” 90) for input to a two-layer perceptron for checking accuracy of classification. This conversion to numeric code was necessary because of the input requirements of ANNs. Comparison between the classification of nonaligned sequences and doubly aligned sequences showed improvement (80% average accuracy for unaligned, 91% average accuracy for doubly aligned), thereby demonstrating the feasibility of the approach [39]. 

Subsequent work [41] reported on a variation to the neural network representation of the doubly aligned sequences. Instead of using ASCII, residues were converted into numerical values for an ANN through real numbers 0.1 to 0.95 in steps of 0.05. This allowed the use of a single layer perceptron, with the ANN returning on average 72% accuracy on nonaligned sequences and 83% on average on doubly aligned sequences. These results demonstrated the sensitivity of the results to ANN architecture (one layer rather than two layers) as well as coding representations. Further work [42] showed the effects of applying different three different amino acid representation methods to virus and worm signatures. The first method was the same as originally used [39, 40], the second method reversed the order of representation, and third shifted the representation by one letter but kept the first letter constant [42] (more details below). Also, the number of signatures was doubled to 60 worm and 60 viral signatures. Accuracy figures showed significant improvement, irrespective of representation method adopted, providing evidence that applying multiple sequencing techniques to malware signatures enhanced predictive capability. 

The aim of this paper is to significantly extend the work started in [42] and to explore the implications of adopting five different residue representations when forming alignments of signatures and extracting motifs. Also, it is important to know whether motifs/metasignatures reported earlier [39, 41, 42] are an accidental by-product of the representations used or evidence of a deeper and unpredicted aspect of applying biosequence techniques to artificial virus and worm signatures. 

## 2. Representations and Methods

There are several tools for alignment and many algorithms used in the study of biosequence analysis. In general, an alignment is an adjustment of a sequence in relation to other sequences. The aim is to arrange two (pairwise alignment) or more (multiple sequence alignment) possibly variable length sequences of DNA or protein in such a way that regions of similarity across sequences (rows of a matrix) fall in the same successive columns of the matrix, where such similarity signifies functional, structural, or evolutionary commonality. Global alignment tries to align every item in every sequence and tends to work best when the sequences are of roughly similar length, such as the Needleman-Wunsch technique [43]. Local alignment, on the other hand, tries to align regions of the sequences even if the sequences are not similar overall, such as the Smith-Waterman technique [44]. ClustalW is a global alignment tool available from the EBI [45] and is the global alignment tool used below. 

Malware is a generic term given to any program or code intended to cause disruption or gain access to unauthorised information and resources. Viruses can be written in any programming language before being compiled. Viral source code signatures are provided on the internet for experimental use and viral source code as such will not be used in this paper. Instead, in line with viral signature detection, the virus signatures, expressed in hexadecimal, are used here. Signature detection is usually effective for new viruses of a known family, where code and functionality are shared and therefore there is some consistency among the signatures to allow or detection of new variants of the same family. The first part of the *virus.1C.Tanga.a* computer virus signature has the hexadecimal coding *8e5ef1aec91259d70c5e62* and the worm *Bat.Agent.bo*, the hexadecimal coding *fb56373bde3881741*. The hexadecimal code for 60 viruses belonging to 12 families and 60 worms belonging to 13 families were downloaded from VX Heavens [46] for use in the experiments below. 

Five different representations of the signatures were tried for alignment purposes (Table 1). The first representation (R1) uses the same order of hexadecimal to amino acid residues. The second (R2) reverses this order and the third (R3) uses a shift of one amino acid residue after the initial residue. R4 essentially swaps the two halves of R1 and R5 reverses the two halves of R1. In previous work, gaps introduced by alignment were coded differently. Here, we use “W” to represent all gaps introduced during the first stage of alignment and “Y” to represent all gaps introduced during the second alignment stage (details below). Given that there are 18! ways to undertake the conversion from hexadecimal plus two gaps into amino acid characters, there is clearly much more work required to assess the effects of different representations. The five chosen here are pseudorandom selections, with no attempt made to ensure lack of random duplication. For instance, hex 5 is represented twice by *F* (R1 and R5). The use of these five representations results in five files, each of 120 instances. The experimental method adopted is as follows.

- Download 60 virus and 60 worm signatures in hexadecimal format from VX Heavens and calculate unaligned benchmarks prior to alignment as follows.
  - Convert the 120 hexadecimal sequences into their five different representation files using Table 1 (R1–R5), resulting in five files of artificial protein sequences (AP1–AP5).
  - Convert these artificial protein sequence representation files (AP1–AP5) into their numeric versions (NR1–NR5) using Table 2 (details below).
  - Input files AP1–AP5 into J48 and Naive Bayes to provide benchmarks for unaligned sequences. Input NR1–NR5 into perceptrons to provide a benchmark for unaligned sequences.
- Input all 60 R1 worm signatures from AP1 (but not virus signatures) into ClustalW to form an initial set of aligned worm sequences. Code gaps as “W.” Repeat for R2–R5 worm signatures. Call these sequences WAR1-WAR5 (“W” for worm, “A” for aligned using ClustalW).
- Input all 60 R1 virus signatures into ClustalW to form an initial set of aligned virus sequences. Code gaps as “W.” Repeat for R2–R5 virus signatures. Call these sequences VAR1–VAR5 (“V” for virus, “A” for aligned using ClustalW).
- Recombine the two aligned sets (WAR1 worm and VAR1 virus) into one dataset (120 sequences of two different lengths) and input into ClustalW to form a second, combined set of aligned sequences, DAR1 (“D” for doubly aligned, “R1” for representation 1). Code all gaps introduced at this (double alignment) stage as “Y.” Repeat for WAR2–WAR5 worm and VAR2–VAR5. This results in five doubly aligned datasets DAR1–DAR5, with each consisting of doubly aligned worm and virus signatures using the same representation.
- Input DAR1–DAR5 to J48 and Naive Bayes. Convert DAR1–DAR5 using Table 2 into their numeric versions (DANR1–DANR5, where “N” is numeric) and input to perceptrons. Compare the results against the benchmarks produced in (a)(iii) above.
- Input DAR1–DAR5 to the rule extractor PRISM to identify virus and worm motifs, or metasignatures.

Whereas previously J48 (a rule extractor with pruning) had been used [39, 41], this paper reports on the application of PRISM (a modular rule extractor [47]) to extract motifs from the signatures. J48 is now used here to provide benchmarks and comparative measures against perceptrons and Naive Bayes because of earlier confidence that J48 works effectively with doubly aligned sequences. J48 and Naive Bayes interpret the input as categorical. However, neural networks require numerical input, hence the conversion to numeric form in Table 2.

Previous work [41] had shown that a single layer perceptron was sufficient but, to take into account the arbitrary nature of the conversion of amino acid residues to numerical values (Table 2), a hidden layer of 72 units was introduced. The hidden layer is intended to collect summed activations from the input layer irrespective of the mode of representation R1–R5 as well as deal with any aspects of nonlinearity due to numerical representation. The hypothesis is that the different representations R1–R5 would make no difference to the training and test results due to the hidden layer acting as a “buffer” between input and output layers. Previous work [41, 43] had shown that a 72 node hidden layer, in comparison to other architectures, was effective. This architecture is used for both benchmarking and comparative purposes on the double aligned sequences. The numeric conversion was used successfully previously [41, 43] and so there is confidence in its effectiveness. The same numeric conversion is used so that any differences in the results can be due only to the mode of representation R1–R5. Class information for supervised learning is attached to the end of each sample as before, with “0” denoting virus and “1” denoting worm. 

Aligning the 60 viruses and 60 worms separately (steps (b) and (c) above) allows the conserved regions of viruses and worms to be independently extracted by ClustalW. ClustalW alignments are based on the frequency of residue occurrence in the 120 input sequences and default weighting parameters based on similarity and dissimilarity of sequences. Without a substitution matrix, ClustalW default parameters use gap insertion and gap extension penalties (e.g., gaps at the ends of sequences are penalised less than gaps in the middle of sequences) as well as protein weight matrices that use similarity of amino acids to each other when calculating where to insert gaps. For the experiments below, it was decided to try the Gonnet substitution matrix [46] with ClustalW. Very generally, Gonnet matrices represent evolutionary substitution information gained from pairwise analysing all protein sequences known in 1992. Current substitution matrices based on exhaustive pairwise alignment tend to focus on specific families of organisms because of the large number of protein sequences now available in public databases. Gonnet matrices have been subsequently refined to take into account growing knowledge of mutations between amino acids. Given that many worm and virus signature sequences are mutated variants of each other, it would be interesting to see how a substitution method based on evolutionary distance would handle the sequences. After the first alignment (steps (b) and (c) above), the length of the virus set alignment will be different from the length of the worm set alignment. This is because there is no guarantee that ClustalW with Gonnet will make the same number of insertions (gaps) for each set of sequences. A second and joint alignment is required to ensure that all 120 sequences are of the same length for machine learning purposes (step (d) above). 

For instance, after alignment by ClustalW, we have (for the first parts of three viral signature sequences only using R1):

 
FIIDIDNGLFDSRPLEEFKGALEGEI…
 
GE-----SQMPSIDMPQF---PGLPS…
 
---------ILHSPMHQFRF-PRSQR…
 
         ::*

which shows that only F is aligned across all three sequences (*) and M and Q across two sequences (:). The gaps (-) introduced at this stage are coded “W.” The 60 aligned sequences for the virus set and the 60 aligned sequences for the worm set were then combined into a composite 120 sequence set for a second alignment. Gaps introduced at this stage are Y gaps. Y and W gaps have their own numeric representation (Table 2). Weka perceptrons were used to implement the neural networks, which has as many input nodes as residues in the fixed length, nonaligned and doubly aligned sequences. (Waikato Environment for Knowledge Analysis: http://www.cs.waikato.ac.nz/ml/weka/). For Weka, each residue position was given its own attribute and the class information was either “virus” or “worm.” J48 and Naive Bayes within Weka were also used for all experiments in this paper. The machine learning task was therefore to determine whether using different representations at the initial stage of encoding worm and virus signatures affected the performance of the perceptrons, J48 and Naive Bayes. For reporting the test results, the following formulae are used (virus is negative; worm is positive):

(1) Accuracy=Number of true positives+number of true negativesNumber of true positives+false positives+false negatives+true negatives,Sensitivity=Number of true positivesNumber of true positives+number of false negatives,Specificity=Number of true negativesNumber of true negatives+number of false positives.

## 3. Experimental Results

The downloaded 60 virus and 60 worm signatures of fixed length 72 hexadecimal characters were first converted into five representation files using R1–R5 (Table 1) and input to Weka perceptrons for benchmark purposes (i.e., without alignment). Previous work had shown that a 72 × 72 × 1 perceptron, with learning rate 0.1 and momentum of 0.25, was sufficient to reduce the root mean squared error to below 0.1 within 150 epochs. A severe training to test ratio of 50 : 50 was used to fully evaluate the generalizability of the three different representations using 10-fold cross-validation as well as test for possible overfitting due to the large number of hidden units. The overall accuracy result for the unaligned sequences was 0.531 (Table 1), which is not much better than tossing a coin. This confirms the problematic nature of the dataset in its raw form. 

The double alignments of worm and virus signatures (steps (b), (c), and (d) above) resulted in fixed length sequences of 140, 123, 128, 133, and 109 for DAR1–DAR5, respectively. These five datasets were converted into numerical input using the coding in Table 2 and input to five perceptrons with architectures 140 × 72 × 1, 123 × 72 × 1, 128 × 72 × 1, 133 × 72 × 1, and 109 × 72 × 1, respectively (step (d) above). The ANN experiment was repeated for 10-folds using the same 50% training, 50% testing regime, and learning parameters as for the benchmark results, leading to the figures displayed in Table 3. Also, DAR1–DAR5 were input to J48 and Naive Bayes in Weka using the same train-test ratios and numbers of folds. The results of the benchmarking (no alignment) and all doubly aligned analysis are provided in Table 3. 

For rule extraction purposes, DAR1–DAR5 were input to PRISM (all samples used for maximum knowledge extraction) to produce the following metasignatures for each representation (where “pos” stands for position in the doubly aligned sequence):

R1: Virus signature if pos5 = A, pos20 = N, pos32 = G or A, pos33 = N or C, pos34 = N, pos60 = C. pos5 = A, pos20 = N, pos21 = D, pos28 = E, pos30 = L, pos32 = A, pos36 = P, pos53 = A.
R1: Worm signature if pos16 = G, pos37 = M, pos93 = I, pos94 = I, pos96 = A, pos100 = C or M, pos104 = D, pos149 = C. pos10 = L, pos41 = C, pos44 = I, pos45 = D or L, pos46 = R, pos51 = H, pos54 = L, pos59 = S, pos70 = G or R, pos71 = S, pos72 = L or M, pos73 = D or P.
R2: Virus signature. No rules found except involving gaps W and Y. pos4 = Q or R, pos43 = K, pos69 = F or Q, pos84 = K.
R2: Worm signature if pos5 = C, pos8 = H, pos11 = C or L, pos27 = G or N, pos28 = D or E, pos43 = A, pos45 = R, pos67 = S. pos5 = C, pos7 = P, pos27 = I, pos28 = D or G, pos29 = C, pos31 = K, pos83 = F.
R3: Virus signature if pos12 = A, post13 = A, pos65 = F.
R3: Worm signature if pos11 = A, pos33 = I, pos55 = L or M, pos88 = M, pos119 = N, pos122 = A, pos124 = H or M.
R4: Virus signature if pos9 = K, pos18 = I, pos21 = K.
 No rules found for virus or worm signatures except those involving W and Y.
R5: Virus signature if pos51 = E or M, pos52 = C, pos53 = P, pos54 = F, pos57 = D, pos58 = F, pos59 = D.
R5: Worm signature if pos13 = H then 1.

Converting these metasignatures back into hexadecimal patterns produces (where “..” means any number of hexadecimal characters and “[  ]” gives alternatives):

R1: Virus signature if “*..2..*[df]..[61][b2]b*..2..*” “..1..b3..4..0..1..c..1..”; Worm signature if “*..6..a..88..1..*[2a]*..3..2..*” “..0..2..8[30]e..7..0..f..[6e]f[0a][3c]*..*”
R2: Virus signature if “..[32]..8..[b3]..8..” Worm signature if “*..6..1..*[67]..[25][5c]*..f..2..1..*” “..e..4..9[ad]e..8..b..”
R3: Virus signature if “*..11..4..*”; Worm signature if “*..1..7..*[90]..0..a..1..[60]*..*”
R4: Virus signature if “..2..1..2..”
R5: Virus signature if “.*.*[6e]*8c5..757..*”; Worm signature if “*..3..*”

## 4. Discussion of Results

Table 3 indicates that the mode of representation affects both unaligned and doubly aligned sequences. The two-layer perceptron performs best on the unaligned sequences (0.562) and Naive Bayes on aligned sequences (0.983) in terms of accuracy. There are major improvements in the results for double aligned sequences, irrespective of representation. The perfect accuracy returned by perceptrons and Naive Bayes on R4 indicates that the insertion of gaps (coded as W and Y) has allowed these two techniques, which use the information present in all attributes including gaps, to distinguish between doubly aligned worm and virus signatures. That is, these two techniques found sufficient information in combinations of attributes (weighted in the case of perceptrons, frequency of occurrence in the case of Naive Bayes) to classify perfectly. J48, however, looks for minimal and selective attributes that distinguish between the two classes. Its performance across all five representations (0.905 average accuracy) is still a major improvement in comparison to unaligned performance (0.527). 

Across the three machine learning algorithms, R5 was best for accuracy and specificity (0.98 and 0.994, resp.), and R3 for sensitivity (0.978). When R1 was used with 60 virus and 60 worm signatures, the metasignatures “..1..b3..4..0..1..1(/c)..1..” for virus and “..0..2..83(/0)e.. 7..0..f..6(/c)fa(/0)3(/c)..” were reported [42]. The results above indicate that the choice of alignment method and use of substation matrix can affect the metasignatures extracted. R1 appears to be best for extracting metasignatures for both virus and worms in terms of information contained in the patterns, followed by R2 and R4 for worm metasignatures only and R5 for virus signatures only. The metasignature for virus using R5 (“.*.*[6e]*8c5..757..*”) in particular contains a number of contiguous hexadecimal characters (no gaps) that could be useful for future AVS to help distinguish viral malware from nonmalware. 

## 5. Conclusions

The results indicate that aligning computer virus and worm signatures using multiple alignment techniques leads to improved classification accuracy using the techniques described in this paper. While the differences in representation are reflected to some extent in classification accuracy after alignment, there is a difference when PRISM is used, with R1 producing more informative metasignatures for both virus and worm. The method of converting malware hexadecimal signatures to residue representation has been clearly demonstrated to affect learning and the motifs extracted. More work is required to determine the tradeoff between representations and richness or usefulness of motifs extracted. Converting the hexadecimal signatures of viruses and worms to amino acids and then rational numbers between 0 to 1 has also been shown to be effective for perceptron learning. Naive Bayes for separating worm from virus signatures after alignment has also been shown to be the most accurate. However, extracting the knowledge contained in Naive Bayes is not easy and symbolic rule extraction techniques are to be preferred when trying to generate malware signatures for scanning files and network packets directly. The derivation of metasignatures provides a new way to look at viral and worm signatures at a “motif” level. These motifs represent common subpatterns among the signatures after initial alignment of virus and worm signatures separately (to allow commonalities among variants of virus and worm signatures to be formed) and then together (to allow differences between virus and worm signatures to be separated). The machine learning task was to separate worm signatures from virus signatures using their hexadecimal form and different amino acid representations, rather than distinguish malware from nonmalware. To test for malware versus nonmalware classification will require mapping the metasignatures back to malware op code, and this is not possible for the signatures used in this research. Nevertheless, we have shown how the growing databanks of malware signatures can be mined for interesting signature information, even if the relationship back to op code is lost or not available. More work is required, however, to identify the most effective alignment algorithms, substitution matrices, and representations for rich and informative metasignatures extraction. 

## Figures and Tables

**Table 1:** Five different representations R1–R5 of malware hexadecimal signatures.

Hex	1	2	3	4	5	6	7	8	9
R1	A	C	D	E	F	G	H	I	K
R2	S	R	Q	P	N	M	L	K	I
R3	A	D	E	F	G	H	I	K	L
R4	I	K	L	M	N	P	Q	R	S
R5	K	I	H	G	F	E	D	C	A

Hex	0	a	b	c	d	e	f	—	—

R1	L	M	N	P	Q	R	S	Y	W
R2	H	G	F	E	D	C	A	Y	W
R3	M	N	P	Q	R	S	C	Y	W
R4	A	C	D	E	F	G	H	Y	W
R5	S	R	Q	P	N	M	L	Y	W

**Table 2:** Conversion of the 16-amino acid alphabet to numeric form between 0 to 1 for input to perceptrons. Y and W (two extra characters) represent the gaps introduced during alignment (see main text).

A	C	D	E	F	G	H	I	K	L	M	N	P	Q	R	S	Y	W
0.1	0.15	0.2	0.25	0.3	0.35	0.4	0.45	0.5	0.55	0.6	0.65	0.7	0.75	0.8	0.85	0.9	0.95

**Table 3:** Results of 50 : 50 train-test ratio, 10-fold cross-validation on all five representations, non-aligned and aligned, using perceptrons, J48 and Naive Bayes.

	Unaligned					Aligned					Unaligned summary	Aligned summary
	R1	R2	R3	R4	R5	R1	R2	R3	R4	R5		
Perceptrons												
Accuracy	0.517	0.533	0.533	0.608	0.617	0.967	0.967	0.975	**1**	0.983	0.562	0.978
Sensitivity	0.516	0.54	0.542	0.607	0.613	0.983	0.967	0.967	**1**	0.968	0.564	0.977
Specificity	0.517	0.529	0.528	0.617	0.633	0.95	0.967	0.983	**1**	1	0.565	0.980
J48												
Accuracy	0.483	0.508	0.558	0.542	0.542	0.825	0.883	0.958	0.883	**0.975**	0.527	0.905
Sensitivity	0.483	0.508	0.554	0.541	0.541	0.783	0.871	**0.982**	0.848	0.967	0.525	0.890
Specificity	0.483	0.509	0.564	0.55	0.55	0.9	0.9	0.933	0.933	**0.983**	0.531	0.930
Naive Bayes												
Accuracy	0.425	0.475	0.533	0.542	0.542	0.975	0.967	0.992	**1**	0.983	0.503	0.983
Sensitivity	0.426	0.476	0.537	0.545	0.545	0.983	0.967	0.984	**1**	0.968	0.506	0.980
Specificity	0.424	0.474	0.53	0.5	0.5	0.967	0.967	1	**1**	1	0.486	0.987
Summary												
Accuracy	0.475	0.506	0.542	0.564	0.567	0.922	0.939	0.975	0.961	**0.98**	0.531	0.955
Sensitivity	0.475	0.508	0.544	0.564	0.566	0.916	0.935	**0.978**	0.949	0.968	0.531	0.949
Specificity	0.475	0.504	0.541	0.556	0.561	0.939	0.945	0.972	0.978	**0.994**	0.527	0.966
